# Integrated Weighted Gene Co-Expression Network and Single-Cell RNA Sequencing Analyses Reveal the Prognostic Significance of Hypoxia in Gastric Cancer

**DOI:** 10.3390/biomedicines14020425

**Published:** 2026-02-13

**Authors:** Chen Jiang, Xingge Li, Yilin Liu, Sicheng Cai, Hailing Yao, Huiying Shi, Kan Wang, Ying Yao, Rong Lin

**Affiliations:** 1 Department of Nutrition, Tongji Hospital, Tongji Medical College, Huazhong University of Science and Technology, Wuhan 430030, China; cheng19950214@163.com; 2 Department of Gastroenterology, Union Hospital, Tongji Medical College, Huazhong University of Science and Technology, Wuhan 430022, China; 3 Department of Traumatology, Tongji Hospital, Tongji Medical College, Huazhong University of Science and Technology, Wuhan 430022, China; 4 Division of Nephrology, Tongji Hospital, Tongji Medical College, Huazhong University of Science and Technology, Wuhan 430030, China

**Keywords:** gastric cancer, hypoxia, single-cell sequencing, weighted gene co-expression network analysis, bioinformatics, prognosis

## Abstract

**Background:** Hypoxia is a key driver of cancer progression. However, its specific prognostic significance in gastric cancer (GC) remains insufficiently characterized. **Methods:** Single-sample gene set enrichment analysis (ssGSEA), weighted gene co-expression network analysis (WGCNA), univariate Cox regression, and least absolute shrinkage and selection operator (LASSO) regression were employed to identify a hypoxia-related prognostic signature. Subsequently, immune microenvironment profiling and single-cell RNA sequencing analyses were employed to further characterize the biological characteristics of the signature. In addition, quantitative real-time polymerase chain reaction (qPCR) was used to validate the expression levels of key hypoxia-associated genes in human GC tissues. **Results:** Elevated hypoxia levels were linked to worse survival outcomes in GC patients. Through integrated WGCNA, Cox, and LASSO analyses, a hypoxia-related prognostic signature (HYS) consisting of four genes—*SPARC*, *AXL*, *NRP1*, and *VCAN*—was established. Patients in the HYS-high group exhibited markedly poorer overall survival than their HYS-low counterparts [*p* = 0.000126, hazard ratio (HR) = 1.936]. Moreover, the HYS-high group exhibited increased infiltration of resting CD4^+^ memory T cells, monocytes, M2 macrophages, and resting mast cells, as well as elevated expression of immunosuppressive molecules, including *PDCD1LG2* and *HAVCR2*. Single-cell RNA sequencing analysis revealed that the signature genes were predominantly expressed in cancer-associated fibroblasts. Consistently, qPCR analysis in five paired GC and para-carcinoma tissues confirmed higher expression of these genes in tumor samples (*p* < 0.01). **Conclusions:** Our findings indicate that hypoxia is a critical determinant of prognosis in GC and is closely associated with an immunosuppressive tumor microenvironment, highlighting its potential value as a prognostic biomarker and therapeutic target.

## 1. Introduction

Globally, gastric cancer (GC) ranks fifth in incidence among all malignancies and is the third most common cause of cancer-related death [1]. Although the five-year survival rate of GC has gradually improved over recent decades, it remains below 30% [2]. Most patients present with advanced-stage disease, where available treatment options have limited efficacy against metastasis, resulting in poor clinical outcomes. Therefore, a comprehensive understanding of the molecular and genetic mechanisms underlying GC is essential for elucidating its pathogenesis, identifying novel therapeutic targets, and ultimately improving patient prognosis.

The tumor microenvironment (TME) constitutes the internal milieu that supports tumor cell survival and progression. TME-related factors, particularly hypoxia, are closely associated with tumor growth, therapeutic resistance, and metastasis [3]. Accumulating evidence indicates that hypoxic microenvironments are a common feature of solid tumors, with hypoxia-related genes frequently enriched in malignancies such as head and neck cancer, lung cancer, and cervical squamous cell carcinoma [4,5]. In recent years, hypoxia has been increasingly recognized as a critical contributor to the initiation and progression of GC [6,7]. However, the role of hypoxia-related genes in specific GC cell subtypes and their prognostic value in GC patients remains not fully elucidated. With the rapid development of public genomic databases, it has become increasingly feasible to systematically identify novel hypoxia-associated biomarkers in GC.

Single-cell RNA sequencing (scRNA-seq) enables the reverse transcription, amplification, and sequencing of the complete transcriptome of individual cells, followed by comprehensive bioinformatic analysis [8]. This technology has been widely applied in cancer research to resolve intratumoral heterogeneity at single-cell resolution. GC exhibits substantial structural and cellular complexity, with malignant cells embedded within a diverse TME composed of epithelial cells, fibroblasts, endothelial cells, and immune cells [9]. The scRNA-seq enables high-resolution analysis of gene expression, allowing accurate identification of diverse cell populations and delineation of intratumoral heterogeneity, thereby facilitating a deeper understanding of how GC heterogeneity influences tumor progression and clinical outcomes. Importantly, scRNA-seq further enables the identification of hypoxic cell subpopulations and hypoxia-associated transcriptional programs within tumor tissues, providing a powerful approach to dissect hypoxia-driven cellular heterogeneity in GC [10]. Although several hypoxia-based prognostic signatures for GC have been proposed using bulk transcriptomic data and integrated single-cell analyses, the cellular origins and biological contexts underlying these signatures remain incompletely characterized [11,12,13]. 

Weighted gene co-expression network analysis (WGCNA) provides a network-based strategy to identify groups of genes with similar expression profiles across multiple samples. This approach clusters highly correlated genes into distinct modules and evaluates their associations with clinical traits or phenotypes, enabling the identification of key biomarker genes and potential therapeutic targets [14]. In the context of hypoxia, WGCNA provides an effective framework to identify co-expressed hypoxia-associated gene modules that are correlated with hypoxia scores and clinically relevant traits, thereby facilitating the systematic prioritization of hypoxia-driven transcriptional programs. Previous studies have applied WGCNA to derive hypoxia-related prognostic models in GC, as well as to characterize gene modules associated with prognosis or molecular subtypes [13,15,16]. However, many of these models rely on relatively large gene sets and lack validation at the single-cell level, limiting their clinical tractability and biological interpretability. In contrast to these studies, our analysis integrates ssGSEA-derived hypoxia scoring to guide module selection and further maps prioritized hypoxia-related modules to specific cellular compartments using single-cell transcriptomic data, thereby providing a cell-type-resolved interpretation of hypoxia-associated gene networks.

In the present study, we integrated ssGSEA-derived hypoxia scoring, WGCNA-based module prioritization, Cox, and LASSO modeling to systematically identify hypoxia-associated prognostic biomarkers in GC. We derived and validated a parsimonious four-gene panel (*SPARC*, *AXL*, *NRP1*, and *VCAN*) across independent cohorts and further mapped its expression to cancer-associated fibroblasts (CAFs) at single-cell resolution. By integrating WGCNA and single-cell transcriptomic analyses, our study provides a cell-type-resolved framework for hypoxia-associated prognostic modeling and offers novel insights into the biological basis of hypoxia-driven GC progression.

## 2. Materials and Methods

### 2.1. Data Processing

**Figure 1:**
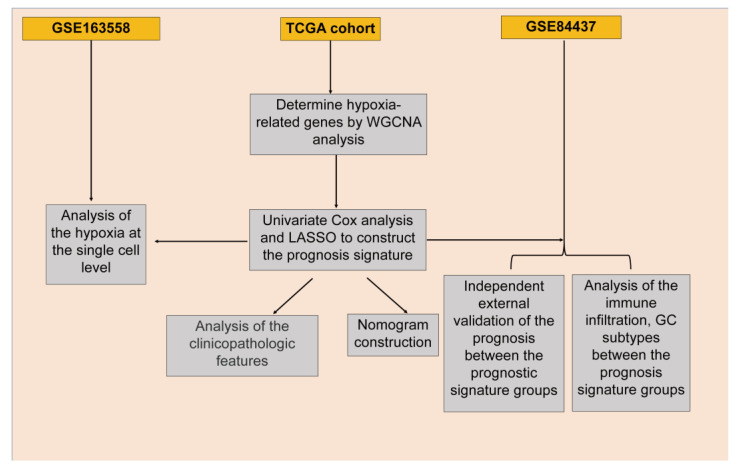
The flowchart of this study.

The GC transcriptomics dataset (GSE84437) and GC single-cell RNA sequencing dataset (GSE163558) were downloaded from the Gene Expression Omnibus (GEO) of the National Center for Biotechnology Information (NCBI) (https://www.ncbi.nlm.nih.gov/geo/) (accessed on 2 February 2026). GSE84437 is a microarray dataset generated on the Illumina HumanHT-12 V3.0 expression beadchip platform (GPL6947). Raw files were processed using standard quantile normalization, followed by log2 transformation, to obtain normalized expression matrices for subsequent analyses. GSE163558 is a single-cell RNA sequencing dataset generated on the Illumina NovaSeq 6000 platform (GPL24676). In addition, the GC cohort from The Cancer Genome Atlas (TCGA) was obtained from the Genomic Data Commons portal (https://portal.gdc.cancer.gov/) (accessed on 2 February 2026) in HTSeq-FPKM format. Genes with low expression (FPKM < 1) in more than 50% of samples were filtered out, and the remaining FPKM values were converted to transcripts per million (TPM), followed by log2(TPM + 1) transformation for subsequent analyses. After data normalization, samples were excluded if overall survival (OS) data were unavailable, key clinicopathological variables (including age or TNM stage) were missing, or the follow-up duration was less than 30 days. Only patients with complete survival data and essential clinical annotations were retained for subsequent analyses. The TCGA-GC cohort was used as the training set, whereas the independent GEO dataset GSE84437 served as an external validation cohort. Consequently, a total of 434 samples from GSE84437, 3 GC samples from GSE163558, and 380 samples from the TCGA dataset were included in the final analyses. The overall workflow of the study is illustrated in Figure 1. 

### 2.2. Single-Sample Gene Set Enrichment Analysis (ssGSEA)

Single-sample GSEA (ssGSEA) was applied via the GSVA R package (v1.48.3) to calculate hypoxia enrichment scores across GC samples, using the MSigDB HALLMARK_HYPOXIA gene set. According to the median ssGSEA score within each cohort (TCGA-GC and GSE84437), GC samples were stratified into high-hypoxia and low-hypoxia groups for subsequent analyses. 

### 2.3. Weighted Co-Expression Network Analysis

WGCNA was performed on the TCGA-GC dataset using the WGCNA package in R to identify stable gene modules associated with hypoxia [10]. In brief, the data were preprocessed by filtering out low-expression genes, and genes with CPM < 1 in more than 50% of samples were removed prior to network construction. The soft-thresholding power (β) was determined following the WGCNA standard procedure by analyzing the scale-free topology fit index across a series of candidate values. The smallest β value achieving approximate scale-free topology (R^2^ > 0.8) with reasonable mean connectivity was chosen. Genes were subjected to clustering to build co-expression networks. These networks were divided into modules, with similar ones being merged. Module–trait relationships were assessed via Pearson correlation analysis between module eigengenes and the hypoxia ssGSEA score, yielding a correlation matrix. Modules characterized by the highest correlation magnitudes with the hypoxia phenotype were carried forward for further investigation.

### 2.4. Gene Enrichment Analysis

Functional enrichment analyses were performed on the hub genes identified from the hypoxia-associated module in the WGCNA analysis. Gene Ontology (GO) and Kyoto Encyclopedia of Genes and Genomes (KEGG) pathway enrichment analyses were conducted using the “clusterProfiler” R package together with the “org.Hs.eg.db” annotation package. Functional terms and pathways were defined as significant when the adjusted FDR was less than 0.05.

### 2.5. Construction of the Prognostic Signature

First, the univariate Cox regression analysis was performed to identify hypoxia-related genes significantly associated with patient prognosis. Subsequently, the LASSO regression algorithm was applied to select the most informative genes and construct the optimal prognostic signature. Patients with GC were divided into high-risk and low-risk categories according to the median hypoxia score derived from the TCGA-GC training set.

### 2.6. Assessment of Prognostic Signature

The predictive accuracy of the hypoxia-related prognostic signature was evaluated using time-dependent receiver operating characteristic (ROC) curve analysis and corresponding area under the curve (AUC) values in both the TCGA-GC training cohort and the independent GEO validation cohort (GSE84437). In addition, clinicopathological features were compared between high- and low-risk groups using the chi-square test for categorical variables and the Wilcoxon rank-sum test for continuous variables. Prognostic determinants were examined through univariate and multivariate Cox models, with hazard ratios (HRs) and 95% confidence intervals (CIs) reported.

### 2.7. Construction of Nomogram

Nomograms were constructed based on the calculated risk scores using the “rms” R package. The nomogram integrated clinicopathological variables, including age, sex, N stage, M stage, and the hypoxia-related prognostic signature, to predict 1-, 3-, and 5-year OS in patients with GC. The predictive performance of the model was evaluated at 1, 3, and 5 years using time-dependent ROC curves implemented in the “timeROC” R package, and calibration analysis was conducted to compare predicted survival estimates with observed outcomes.

### 2.8. Immune Infiltration Analysis

Immune cell infiltration in GC samples from the GEO cohort (GSE84437) was evaluated to characterize the tumor immune microenvironment and the functional relevance of immune-related genes. The relative proportions of 22 infiltrating immune cell subsets were estimated using the CIBERSORT algorithm with the LM22 signature matrix and 1000 permutations. Only samples with CIBERSORT output *p* < 0.05 were retained for subsequent analyses to ensure reliable deconvolution results. The distribution of immune cell fractions across samples, as well as the correlations among different immune cell populations, were visualized using ggplot2 (version 3.4.4) and pheatmap (version 1.0.12) R packages. Immune infiltration patterns and immune checkpoint expression across risk groups were compared using a violin plot visualization.

### 2.9. Consensus Clustering Analysis of Hypoxia-Related Genes

Consensus clustering analysis was performed using the ConsensusClusterPlus R package to classify GC samples into distinct molecular subtypes based on the expression profiles of hypoxia-related genes. The number of clusters (k) was tested from 2 to 9 using the partitioning around medoids (PAM) method with 1 − Pearson correlation as the distance metric. The optimal number of clusters (k = 3) was selected based on the delta area plots and the stability of the consensus heatmaps. Batch effects were adjusted prior to clustering to minimize technical bia

### 2.10. Single-Cell RNA Sequencing Analysis

Raw sequencing data of the GSE163558 dataset were initially processed using Cell Ranger (version 6.1.2) for read alignment, barcode processing, and generation of the gene–cell count matrices. Downstream analyses were performed using the Seurat R package (version 4.3.0). Cells were filtered out if they expressed fewer than 400 genes or more than 5000 genes, or if the proportion of mitochondrial gene expression exceeded 10% or erythrocyte gene expression exceeded 3%. After quality control, data normalization, scaling, and identification of highly variable genes were conducted in Seurat, and genes with the highest variability (n = 1500) were carried forward for downstream analyses. Principal component analysis (PCA) was performed on the scaled data, and the top 20 principal components were used for downstream clustering. Louvain clustering (resolution 0.5) was used to define cell populations, which were visualized using t-SNE. Marker genes were identified for each cluster using FindAllMarkers with the thresholds of |log2 fold change| > 0.25, minimum percentage of expressing cells (min.pct) > 0.25, and adjusted *p* value < 0.05 (Supplementary File S1). Cell-type annotation was performed using the SingleR package with the Human Primary Cell Atlas reference dataset, resulting in the identification of 12 clusters that were further grouped into seven major cell types. The genes for each cluster were further analyzed and visualized using heatmaps, t-SNE plots, and bubble plots. 

### 2.11. Pseudotime Analysis

CAFs were operationally defined as the stromal cluster annotated based on canonical fibroblast or ECM markers. All CAF-annotated cells retained after quality control were extracted for trajectory inference (n = 238). Pseudotime analysis was conducted using the Monocle R package, in which ordering genes were selected as highly variable genes or cluster marker genes within the CAF subset, followed by dimensionality reduction using the DDRTree method and cell ordering along the reconstructed trajectory.

### 2.12. Quantitative Real Time-PCR (qRT-PCR)

GC tissues and paired para-carcinoma tissues were obtained from five patients at the Endoscopy Center of Wuhan Union Hospital with written informed consent from all participants. The study protocol was approved by the Institutional Review Board of Tongji Medical College, Huazhong University of Science and Technology (IORG No. IORG0003571). Total RNA was extracted using TRIzol (Vazyme, Nanjing, China), and RNA integrity was assessed by spectrophotometry, with samples showing A260/A280 values between 1.8 and 2.0 selected for downstream analyses. For reverse transcription, 1 μg of total RNA was reverse-transcribed into cDNA using a commercial reverse transcription kit (Vazyme, China). Gene expression was quantified by qRT-PCR using SYBR Premix Ex Taq (Vazyme, China). Each 20 μL reaction contained SYBR Premix, gene-specific primers, diluted cDNA, and nuclease-free water. The amplification protocol included an initial denaturation at 95 °C for 30 s, followed by 40 cycles of 95 °C for 5 s and 60 °C for 30 s. Specificity was confirmed by melt curve analysis. The expression values were normalized to *GAPDH* using the 2^−ΔΔCt^ approach. Primer sequences used in this study are listed in Supplementary File S2.

### 2.13. Statistical Analysis

All statistical analyses were performed using R software (version 4.2.1) and GraphPad Prism (version 6.0; GraphPad Software, San Diego, CA, USA). Data normality was assessed before applying parametric tests. Differences between the two groups were evaluated using Student’s *t*-test for normally distributed variables and the Wilcoxon rank-sum test for non-normally distributed variables. For multiple comparisons in high-dimensional analyses, FDR correction was applied using the Benjamini–Hochberg method. OS was defined as the time from initial diagnosis to death or last follow-up. Survival analyses were conducted using the Kaplan–Meier (K–M) method, with group differences compared by the log-rank test. HRs and 95% CIs were derived from Cox regression models, and the proportional hazards assumption was verified via Schoenfeld residuals. The prognostic performance of the hypoxia-related signature was further evaluated using time-dependent ROC analysis and concordance indices. *p* value < 0.05 was considered statistically significant.

## 3. Results

### 3.1. Screening for Genes Associated with the Hypoxia Phenotype by WGCNA

**Figure 2:**
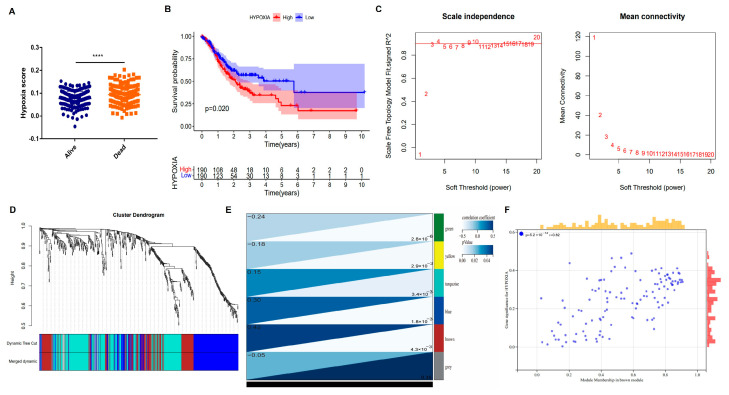
ssGSEA analysis and weighted co-expression network analysis: (**A**) Hypoxia score was higher in the dead GC patients than in alive GC patients. (**B**) K-M survival analysis showed that patients in the high-hypoxia group experienced poorer OS. (**C**) Scale-free topology model fit (**left**) and mean connectivity (**right**) across a range of soft-thresholding power values. The numbers (1–20) indicate the corresponding soft-thresholding power used in network construction. (**D**) Dynamic hierarchical clustering. Different colors represented different gene modules; the dynamic tree cut corresponded to the initially obtained gene module, and the merged dynamic corresponded to the final merged gene module. (**E**) Correlation heatmap showing the relationships between module eigengenes and clinical characteristics, annotated with correlation values and *p* values. (**F**) Scatter plot depicting the relationship between module membership (MM) and gene significance (GS) for genes within the brown module. The yellow and pink marginal histograms represent the distributions of MM and GS values, respectively. **** *p* < 0.0001.

According to the median hypoxia enrichment score, GC patients were divided into high- and low-hypoxia groups. We found that deceased patients exhibited significantly higher hypoxia enrichment scores than surviving patients (Figure 2A). Consistently, in the TCGA cohort, patients in the high-hypoxia group had a significantly shorter OS compared with those in the low-hypoxia group (*p* < 0.05, Figure 2B). For WGCNA construction, a scale-free topology fitting index (R^2^) exceeding 0.8 was used, and the soft-thresholding power (β) was set to 3 (Figure 2C). After filtering low-expression genes, a total of 8,421 genes were included for WGCNA network construction. The hierarchical cluster tree identified six co-expression modules after merging highly similar modules. Module–trait relationship analysis revealed that the brown module, containing 161 genes, showed the strongest positive correlation with the hypoxia phenotype (correlation = 0.42, *p* < 0.001, Figure 2D,E). The intramodular genes within the brown module were further prioritized based on stringent criteria of module membership (MM > 0.9) and gene significance (GS > 0.9) to identify a high-confidence set of candidate hub genes (Figure 2F). Using these thresholds, 161 putative hub genes were retained for subsequent analyses.

### 3.2. Enrichment Analysis

**Figure 3:**
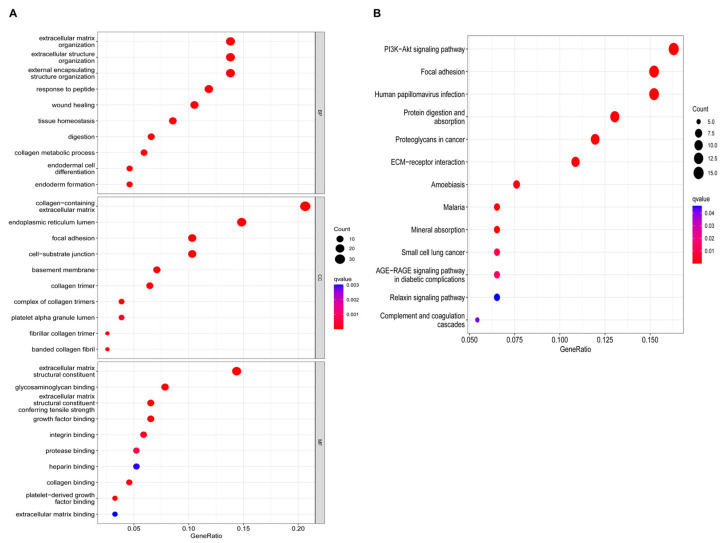
Enrichment analysis: (**A**) Gene Ontology (GO) enrichment. (**B**) Kyoto Encyclopedia of Genes and Genomes (KEGG) enrichment.

The 161 genes belonging to the brown module identified by WGCNA were subjected to functional enrichment analysis. GO terms were explored within the biological process (BP), cellular component (CC), and molecular function (MF). In the BP category, the hypoxia-associated co-expressed genes were predominantly enriched in extracellular matrix organization, extracellular structure organization, and external encapsulating structure organization. For CC, these genes were mainly associated with the collagen-containing extracellular matrix, endoplasmic reticulum lumen, focal adhesion, and cell–substrate junction. In the MF category, enrichment was primarily observed in extracellular matrix structural constituents and glycosaminoglycan binding (Figure 3A). KEGG enrichment analysis revealed that the hypoxia-associated co-expressed genes were primarily enriched in pathways related to PI3K–Akt signaling, focal adhesion, human papillomavirus infection, protein digestion and absorption, and proteoglycans in cancer (Figure 3B). 

### 3.3. Construction of the Prognostic Signature Associated with Hypoxia

**Figure 4:**
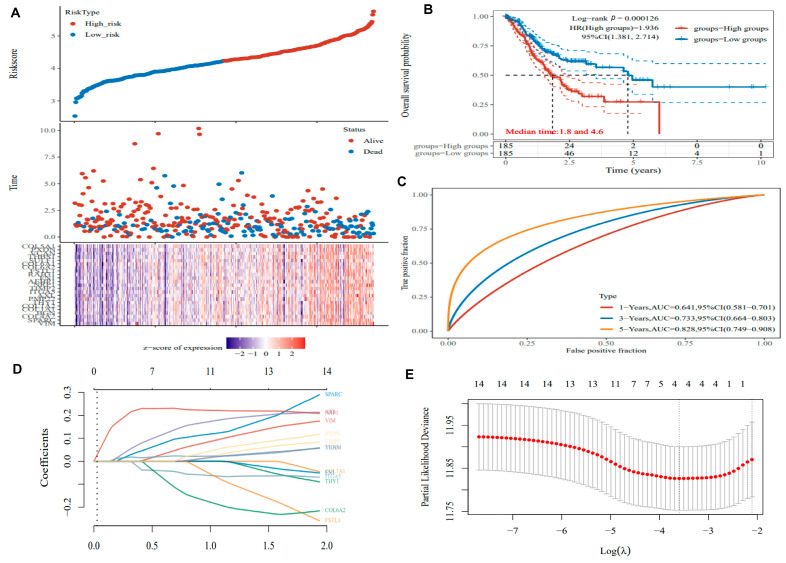
Construction of the hypoxia-related prognostic signature.: (**A**) Risk score distribution, survival outcomes, and gene expression profiles across risk groups in the TCGA dataset. (**B**) Kaplan–Meier survival curves comparing survival between high- and low-risk patients. (**C**) ROC curve of the risk model. (**D**,**E**) Key prognostic genes were screened using LASSO Cox regression.

As shown in Figure 4, the univariate Cox regression model was employed to evaluate the prognostic relevance of hypoxia-related genes. The distribution of gene expression levels, ordered from low to high risk, is shown in the upper panel, together with the corresponding survival status and survival time presented as scatter plots. The lower panel displays a heatmap of gene expression patterns. Compared with the low-hypoxia risk group, patients in the high-hypoxia risk group exhibited a markedly higher mortality rate (Figure 4A). According to the K-M survival analysis, the OS of GC patients in the high-hypoxia group was shorter than that of the low-hypoxia group [*p* < 0.001, HR = 1.936, 95% CI (1.381, 2.714), Figure 4B]. The median survival time corresponding to a 50% survival probability was 1.8 years in the high-hypoxia group and 4.6 years in the low-hypoxia group (Figure 4B). The predictive performance of the hypoxia-related prognostic model was further evaluated using time-dependent ROC analysis in the TCGA training cohort, yielding AUC values of 0.641 (95% CI: 0.581–0.701) for 1-year OS, 0.733 (95% CI: 0.664–0.803) for 3-year OS, and 0.828 (95% CI: 0.749–0.908) for 5-year OS (Figure 4C). 

Subsequently, the Lasso regression analysis was applied to further refine the prognostic gene set, and the final model coefficients were derived from the TCGA training cohort. Four genes—*SPARC*, *AXL*, *NRP1*, and *VCAN*—were identified as the most robust prognostic markers and were used to construct the hypoxia-related prognostic signature. The hypoxia-related gene signature (HYS) was derived based on the following weighted formula: HYS = (0.0241 × *SPARC*) + (0.0474 × *AXL*) + (0.2065 × *NRP1*) + (0.0168 × *VCAN*) (Figure 4D,E). Based on the median HYS risk score, GC patients were stratified into high-risk HYS and low-risk HYS groups for subsequent analyses. 

### 3.4. Assessment of the Prognostic Value of the Hypoxia Gene Signature

**Figure 5:**
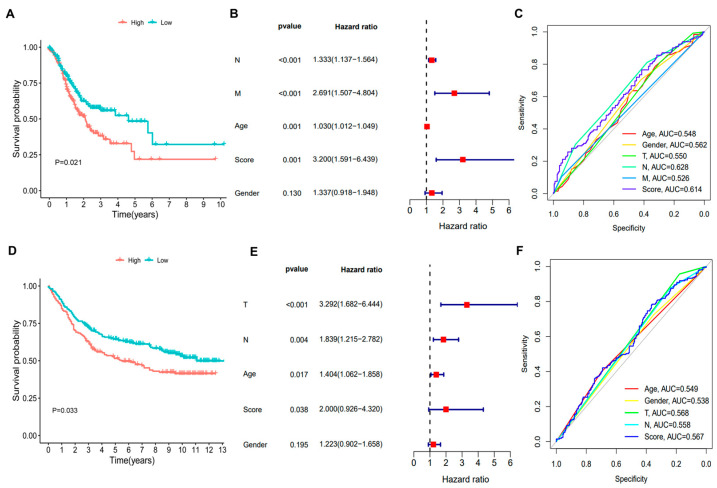
Assessment of the prognosis of the hypoxia gene signature in TCGA and GEO datasets: (**A**) Kaplan–Meier survival analysis of the hypoxia gene signature in the TCGA cohort. (**B**) Multivariate Cox analysis in the TCGA cohort. (**C**) AUC of HYS and clinicopathological characteristics in the TCGA cohort. (**D**) Kaplan–Meier survival analysis of hypoxia gene signature in GSE84437. (**E**) Multivariate Cox analysis in GSE84437. (**F**) AUC of HYS and clinicopathological characteristics in GSE84437.

To further validate the predictive accuracy of the hypoxia gene signature, prognostic performance was evaluated in both the TCGA training cohort and the GEO validation cohort (GSE84437). The survival curve revealed that patients in the high-risk HYS group had significantly shorter OS compared with those in the low-risk HYS group in both the TCGA cohort (*p* = 0.021, Figure 5A) and the GSE84437 cohort (*p* = 0.033, Figure 5D). In the TCGA cohort, multivariate Cox regression analysis incorporating the HYS risk score and clinicopathological variables, including age, sex, N stage, and M stage, demonstrated that the HYS risk score remained an independent prognostic factor (HR = 3.200, 95% CI: 1.591–6.439, *p* = 0.001), together with N stage (HR = 1.333, 95% CI: 1.137–1.564, *p* < 0.001), M stage (HR = 2.691, 95% CI: 1.507–4.804, *p* < 0.001), and age (HR = 1.030, 95% CI: 1.012–1.049, *p* = 0.001) (Figure 5B). In the independent GSE84437 cohort, multivariate Cox analysis incorporating the HYS risk score and clinicopathological variables, including age, sex, T stage, and N stage, confirmed that the HYS risk score remained independently associated with OS (HR = 2.000, 95% CI: 0.926–4.320, *p* = 0.038), together with T stage (HR = 3.292, 95% CI: 1.682–6.444, *p* < 0.001), N stage (HR = 1.839, 95% CI: 1.215–2.782, *p* = 0.004), and age (HR = 1.404, 95% CI: 1.062–1.858, *p* = 0.017) (Figure 5E). Furthermore, ROC curve analysis indicated that these clinicopathological variables exhibited consistent prognostic value in predicting survival outcomes of GC patients (Figure 5C,F).

### 3.5. Prognostic Value of the Hypoxia Signature Across Different Clinicopathological Subgroups

**Figure 6:**
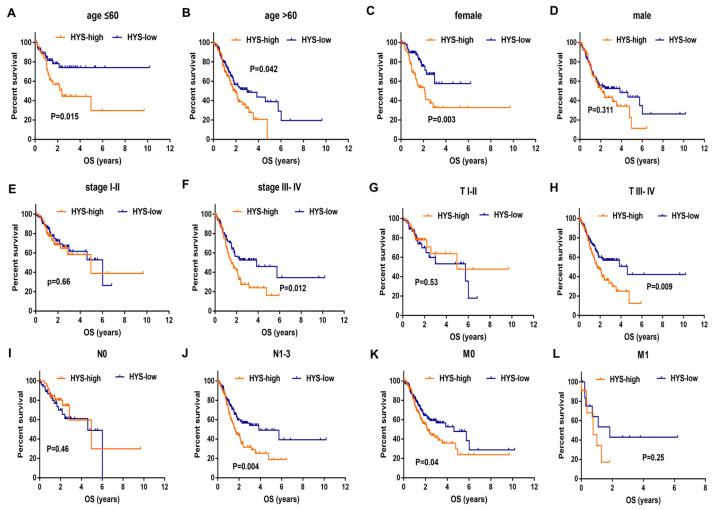
Kaplan–Meier curves for overall survival in HYS-high group and HYS-low group according to (**A**,**B**) age, (**C**,**D**) gender, (**E**,**F**) stage, (**G**,**H**) T stage, (**I**,**J**) N stage, (**K**,**L**) M stage.

K-M survival analyses were performed in the TCGA cohort to evaluate the prognostic applicability of the hypoxia signature across different clinicopathological subgroups (Figure 6A–L). The results showed that GC patients in the HYS-low group consistently exhibited longer OS than those in the HYS-high group across multiple stratifications, including age (≤60 years, *p* = 0.015; >60 years, *p* = 0.042), sex (female, *p* = 0.003), TNM stage (stage III–IV, *p* = 0.012), T stage (T3–4, *p* = 0.009), N stage (N1–3, *p* = 0.004), and M stage (M0, *p* = 0.040). After FDR correction, patients in the high-risk HYS group exhibited significantly shorter OS in several clinically relevant subgroups, including patients aged ≤60 years (adjusted *p* = 0.036), female patients (adjusted *p* = 0.024), patients with advanced stage (III–IV, adjusted *p* = 0.036), patients with higher T stage (T3–4, adjusted *p* = 0.036), and patients with higher N stage (N1–3, adjusted *p* = 0.024).

### 3.6. Predictive Nomogram for Prognosis in Patients with GC

**Figure 7:**
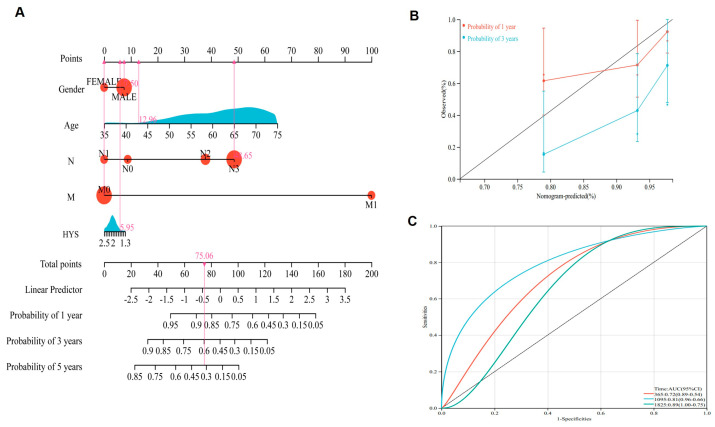
Predictive nomogram for prognosis in patients with GC: (**A**) Nomogram analysis was performed to estimate 1-, 3-, and 5-year survival probabilities in GC patients. (**B**) Calibration plots illustrating the agreement between predicted and observed survival at 1 and 3 years. (**C**) ROC curves and corresponding AUC values assessing the prognostic performance of the nomogram.

According to the risk scores and clinicopathological variables, including age, gender, N stage, M stage, and HYS, a prognostic nomogram was constructed to predict 1-, 3-, and 5-year OS in patients with GC (Figure 7A). The nomogram demonstrated good discriminative performance, with a C-index of 0.730 (95% CI: 0.630–0.821; *p* < 0.001). Meanwhile, the calibration analysis indicated favorable agreement between the predicted and observed survival probabilities at 1 and 3 years (Figure 7B). In addition, the ROC curve analysis showed that the nomogram achieved AUC values of approximately 0.72, 0.81, and 0.89 for predicting 1-, 3-, and 5-year survival, respectively (Figure 7C).

### 3.7. Immune Infiltration Analysis Between HYS-High and HYS-Low Groups

**Figure 8:**
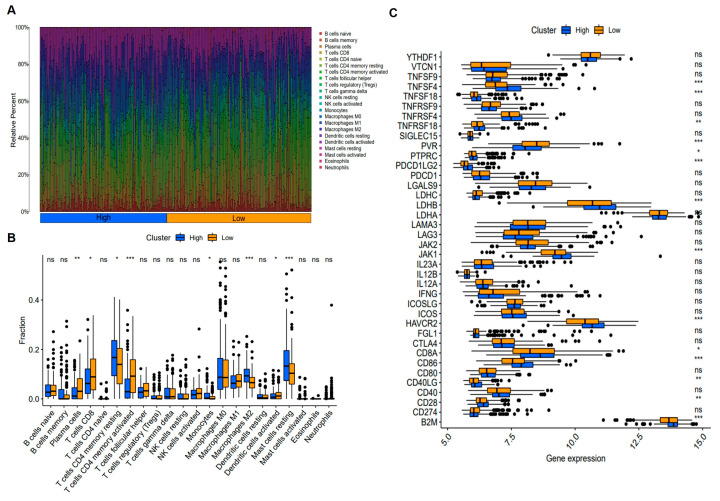
Immune infiltration analysis (**A**) Overview of the immune landscape in patients with gastric cancer. (**B**) Comparison of immune cell infiltration levels between the HYS-high and HYS-low groups. (**C**) Immune checkpoint gene expression profiles stratified by HYS status. * *p* < 0.05, ** *p* < 0.01, *** *p* < 0.001, ns: not significant.

The immune composition of the GC microenvironment was systematically evaluated through immune infiltration analysis. The overall immune landscape of GC is illustrated in Figure 8A, revealing that T cells constituted the predominant immune cell population within tumor tissues. Subsequently, immune cell infiltration levels were compared between the HYS-high and HYS-low groups. As shown in Figure 8B, the HYS-high group exhibited significantly higher proportions of resting CD4^+^ memory T cells (*p* < 0.05), monocytes (*p* < 0.05), M2 macrophages (*p* < 0.001), and resting mast cells (*p* < 0.001). In contrast, the HYS-low group showed increased infiltration of plasma cells (*p* < 0.01), CD8^+^ T cells (*p* < 0.05), activated CD4^+^ memory T cells (*p* < 0.001), and activated dendritic cells (*p* < 0.05). Immune checkpoints contribute to an immunosuppressive TME by dampening effective anti-tumor immune responses. Expression analysis revealed that *TNFSF4*, *TNFRSF9*, *TNFRSF18*, *PTPRC*, *PDCD1LG2*, *LDHB*, *JAK1*, *HAVCR2*, *CD86*, *CD40LG*, and *CD28* were markedly elevated in the HYS-high group. Conversely, *PVR*, *CD8A*, and *B2M* were more highly expressed in the HYS-low group (Figure 8C). 

### 3.8. Consensus Cluster Analysis of Hypoxia-Related Genes

**Figure 9:**
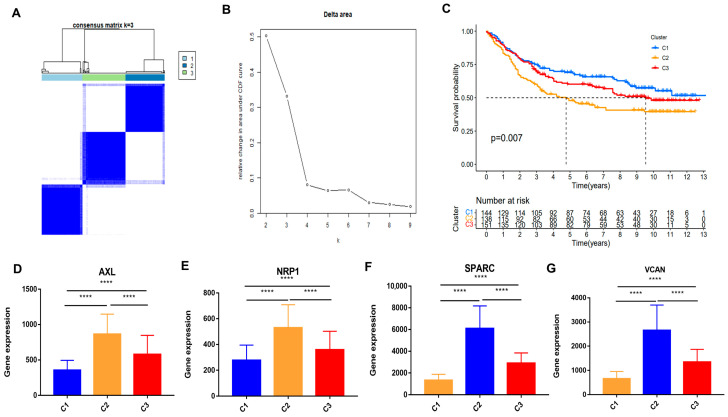
Consensus clustering of GC in GSE84437: (**A**) Consensus clustering matrix of gastric cancer samples at k = 3. (**B**) Delta area curve illustrating changes in clustering stability with k = 3. (**C**) Kaplan–Meier survival curves for the three identified clusters. (**D**–**G**) Expression patterns of *AXL*, *NRP1*, *SPARC*, and *VCAN* across the three clusters. **** *p* < 0.0001.

Using the expression profiles of the hypoxia-associated genes *AXL*, *NRP1*, *SPARC*, and *VCAN*, consensus clustering was conducted on 434 GC samples from GSE84437. Using this approach, the samples were classified into three distinct molecular subtypes, designated cluster 1 (C1), cluster 2 (C2), and cluster 3 (C3) (Figure 9A,B). OS varied significantly across the three clusters, as shown by K–M survival curves (log-rank *p* = 0.007, Figure 9C). Patients in C2 exhibited the poorest prognosis, whereas those in C1 showed the most favorable survival outcome. Notably, patients in C3 demonstrated an intermediate survival pattern between C1 and C2. Consistent with these survival trends, expression analysis demonstrated that *AXL*, *NRP1*, *SPARC*, and *VCAN* were highly expressed in C2, moderately expressed in C3, and lowly expressed in C1, indicating a progressive hypoxia-associated expression pattern across the three clusters (Figure 9D–G).

### 3.9. Expression of Hypoxia-Related Genes in Single Cells of GC

**Figure 10:**
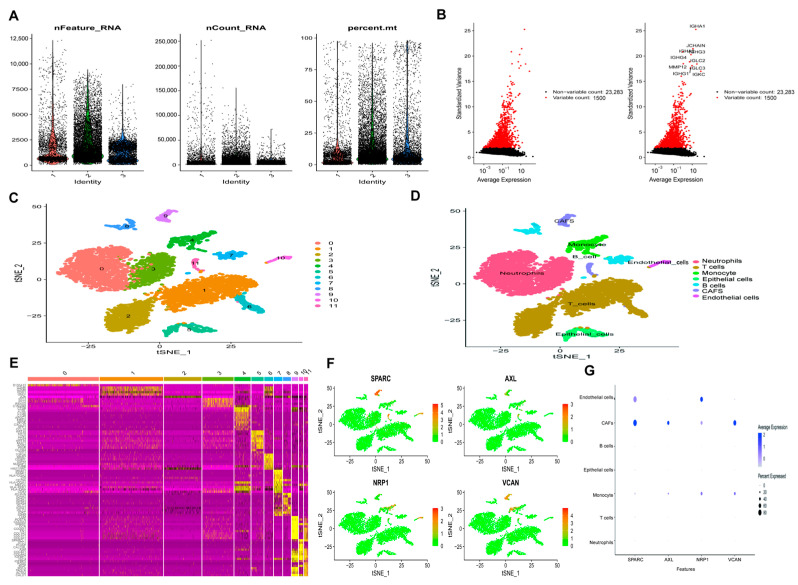
Single-cell analysis: (**A**) Filtering based on mitochondrial gene content was applied to ensure cell quality. (**B**) Identification of highly variable genes, with the top 1500 features highlighted in red and the top 10 genes annotated. (**C**) Dimensionality reduction and clustering analysis of the GC dataset, revealing 12 distinct cell clusters. (**D**) The 12 clusters can be divided into B cells, T cells, epithelial cells, endothelial cells, CAFs, monocytes, and neutrophils. (**E**) Cell cluster gene expression heatmap. Yellow indicates higher expression and purple indicates lower expression. (**F**) The genes expressed in different cell clusters. (**G**) The gene expression levels of different cell clusters are displayed.

The quality control analysis and dimension reduction clustering of the single-cell dataset (GSE163558) are presented in Figure 10. Initially, quality control metrics, including RNA count, gene count, and mitochondrial gene percentage, were evaluated to ensure data reliability (Figure 10A). The top 1500 variable genes were then identified and are highlighted in red, with the top 10 genes labeled (Figure 10B). Dimensionality reduction followed by clustering analysis classified the cells into 12 discrete groups. According to cluster-specific marker genes, these clusters were further annotated into seven major cell types, namely B cells, T cells, epithelial cells, endothelial cells, CAFs, neutrophils, and monocytes (Figure 10C,D). To define cluster marker genes, differential gene expression analysis was conducted between each cluster and all remaining cells (Figure 10E). The distribution of hypoxia-related genes across different cell types was visualized using feature plots and dot plots, which reflect both average expression levels and the proportion of expressing cells (Figure 10F,G). The signature genes *SPARC*, *AXL*, *VCAN*, and *NRP1* showed relative enrichment in CAFs compared with other major cell populations, while *NRP1* also exhibited notable expression in endothelial cells, suggesting cell-type-specific involvement of hypoxia-related pathways in GC.

### 3.10. Pseudotime Analysis of Hypoxia-Related Genes in Tissue Stem Cells

**Figure 11:**
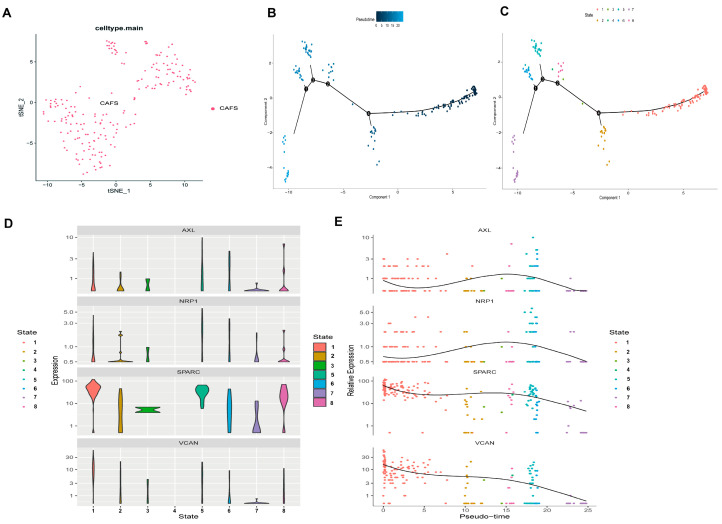
Pseudotime analysis of hypoxia-related genes in CAFs: (**A**) All cells analyzed were CAFs. (**B**) Differences in cell differentiation times. (**C**) The eight differentiation states of CAFs. (**D**) Gene expression levels across different cellular states. (**E**) Dynamic gene expression changes during CAF differentiation. Each dot representing a single cell colored according to its corresponding cell state.

The pseudotime analysis was conducted on CAFs to investigate the dynamic relationship between cellular differentiation and the expression patterns of the four hypoxia-related signature genes. As shown in Figure 11A, all cells included in the trajectory analysis were annotated as CAFs. In Figure 11B, the color gradient represents pseudotime progression, with CAFs transitioning from the right (deep blue) to the left (light blue) along the differentiation trajectory. Notably, the differentiation process of CAFs was partitioned into eight distinct differentiation states, each represented by a unique color (Figure 11C). The expression levels of the hypoxia-related signature genes across these states are illustrated in Figure 11D. Along pseudotime, *AXL* and *NRP1* showed modest, non-monotonic expression changes, whereas *SPARC* and *VCAN* exhibited a gradual decreasing tendency (Figure 11E).

### 3.11. Expression of the Four Signature Genes in GC and Para-Carcinoma Tissues

**Figure 12:**
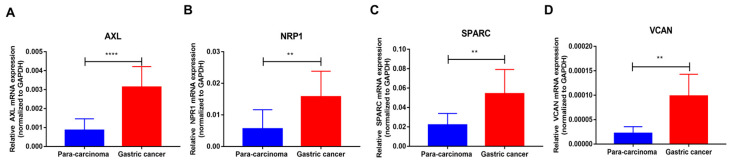
The expression of four genes in GC and para-carcinoma tissues. **** *p* < 0.0001, ** *p* < 0.01. (**A**) *AXL*; (**B**) *NRP1*; (**C**) *SPARC*; (**D**) *VCAN*.

To provide experimental support for the hypoxia-related signature, qRT-PCR was performed in five paired GC and para-carcinoma tissues. Compared with matched para-carcinoma tissues, *AXL*, *NRP1*, *SPARC*, and *VCAN* consistently exhibited higher expression levels in GC samples (*p* < 0.01, Figure 12A–D). In addition, we examined differences in the expression of four hypoxia-associated genes between TCGA GC samples and normal gastric tissues from the GTEx cohort. As shown in Supplementary Figure S1, *AXL*, *NRP1*, *SPARC*, and *VCAN* were all significantly upregulated in GC tissues compared with normal gastric mucosa (all *p* < 0.001).

## 4. Discussion

GC, one of the most common malignant tumors worldwide, poses a substantial burden on public health and significantly reduces life expectancy. Rapid tumor cell proliferation combined with insufficient or delayed angiogenesis often leads to inadequate blood perfusion within tumor tissues, thereby promoting the formation of a hypoxic TME. In recent years, tumor hypoxia has emerged as a central focus in cancer research, given its pivotal involvement in cancer progression, metastasis, therapeutic resistance, prognosis, and treatment responsiveness [17]. Despite its importance, hypoxia-related gene signatures for accurately predicting prognosis in GC remain poorly characterized. In recent years, several hypoxia-related gene signatures have been proposed for prognostic stratification in GC. However, their biological interpretability, cell-type specificity, and clinical tractability remain incompletely elucidated.

In the present study, hypoxia emerged as an independent prognostic risk factor in GC, with patients exhibiting higher hypoxia ssGSEA scores showing significantly reduced overall survival compared with those with lower scores. Using WGCNA, we identified gene modules most strongly associated with hypoxia and subsequently established a hypoxia-related prognostic gene signature through LASSO regression and multivariate Cox analysis. The stability of the prognostic signature was confirmed across both TCGA and GEO cohorts, with high-risk HYS patients consistently exhibiting inferior survival compared with those in the low-risk group. ROC analysis further supported the hypoxia-related gene signature as having a moderate clinically meaningful ability to predict prognosis in GC. Subgroup analyses revealed that the adverse prognostic impact of hypoxia was particularly evident across multiple clinicopathological strata, including patients aged ≤60 years, female patients, those with advanced disease (stage III–IV), higher T stage (T3–4), nodal involvement (N1–3), and non-metastatic status (M0). Moreover, *AXL, NRP1, SPARC*, and *VCAN* were significantly upregulated in GC tissues compared with paired para-carcinoma samples. Given that para-carcinoma tissues may not represent truly normal mucosa, additional validation using GTEx normal gastric tissues confirmed consistent overexpression of these genes in GC, supporting the robustness of our results. Collectively, these findings underscore the important role of hypoxia in GC progression and demonstrate the potential clinical relevance of hypoxia-associated molecular signatures in prognostic stratification.

The four genes comprising the hypoxia-related signature (*SPARC, AXL, VCAN,* and *NRP1*) have all been implicated in GC progression and tumor–stromal interactions. *SPARC*, a stromal cell-associated glycoprotein, has been reported to inhibit GC cell proliferation by inducing apoptosis, and its downregulation is associated with improved prognosis in GC patients [18,19]. *AXL*, a receptor tyrosine kinase, plays a pivotal role in tumor–stromal interactions. Bae et al. demonstrated that inhibition of the *GAS6/AXL* axis suppresses tumor progression by disrupting the crosstalk between GC-associated fibroblasts and cancer cells [20]. As a major component of the extracellular matrix, *VCAN* has emerged as a prognostically relevant molecule in GC and is implicated in promoting tumor growth and invasion [21]. In addition, *NRP1*, a multifunctional co-receptor, has been reported to facilitate GC growth and migration [22]. In line with these reports, our single-cell analysis suggested that these genes are relatively enriched in CAFs, with *NRP1* also exhibiting notable expression in endothelial cells. These observations support the notion that hypoxia-associated transcriptional programs may be preferentially engaged within stromal components of the GC microenvironment. Pseudotime analysis further suggested gradual changes in the expression of hypoxia-related genes along inferred CAF state transitions, with *SPARC* and *VCAN* exhibiting a gradual decline, while *AXL* and *NRP1* displayed a characteristic down–up–down expression trend.

A heterogeneous population of innate and adaptive immune cells within the TME is pivotal to the development and advancement of GC [23]. Immune infiltration analysis in our study demonstrated that T cells constituted the dominant immune cell population within GC tissues. Previous studies have reported that the abundance of CD4^+^ naive T cells, CD4^+^ memory T cells, CD8^+^ T cells, and activated CD8^+^ T cells increases with tumor progression, highlighting the pivotal role of T cell-mediated immunity in cancer development [24]. Notably, we further observed a significantly higher proportion of M2 macrophages in the high-risk HYS group. M2-polarized macrophages are known to exert pro-tumorigenic effects by promoting tumor growth, angiogenesis, and metastasis, thereby contributing to an immunosuppressive microenvironment and unfavorable clinical outcomes [25]. 

Immune checkpoint molecules can suppress anti-tumor immune responses and facilitate immune evasion by tumors through their interactions with cognate ligands or receptors on cancer cells [26,27]. Recently, several key immune checkpoint molecules have been identified as promising immunotherapeutic targets, and immune checkpoint inhibitors have been widely implemented in clinical oncology. Our analyses revealed a close link between hypoxic status and immune checkpoint profiles, distinguishing the high-risk HYS group from the low-risk group. Notably, *PDCD1LG2* (*PD-L2*), a critical immunosuppressive molecule that inhibits immune-mediated tumor cell killing, was significantly upregulated in the high-risk HYS group. Previous studies have shown that PD-1/PD-L2 interactions play a functional role in suppressing anti-tumor immune responses within the GC-TME [28,29]. These results provide a possible explanation for the adverse prognostic impact of heightened hypoxia in GC.

Some limitations should be considered when interpreting these findings. First, the qRT-PCR validation was exploratory and focused on a limited number of candidate genes with a small sample size. Therefore, multiple-testing correction was not applied. These results should be interpreted with caution and require confirmation in larger, independent clinical cohorts and additional control genes. Second, the single-cell RNA sequencing analysis was based on only three GC samples, which limits generalizability and precludes a robust assessment of inter-patient heterogeneity. Third, although the proposed hypoxia-related prognostic signature demonstrated statistically significant prognostic value, it was not directly benchmarked against previously published GC prognostic models or advanced machine learning-based survival algorithms. As a result, its relative performance compared with established gene signatures or nonlinear survival models remains to be determined. In addition, the gene selection strategy employed in this study, integrating WGCNA module membership, Cox regression, and LASSO shrinkage, primarily identifies co-expressed genes with linear associations to survival outcomes. While this framework enhances interpretability and clinical applicability, it does not explicitly model condition-specific differential expression or nonlinear feature importance and higher-order interactions. Future studies incorporating complementary differential expression analyses and machine learning-based survival modeling approaches may further refine feature prioritization and deepen mechanistic interpretation.

In conclusion, we systematically analyzed GC data from public databases to construct a prognostic signature of hypoxia-related genes. Using the median signature score, patients were stratified into high-risk HYS and low-risk HYS subgroups, with consistently poorer survival outcomes observed in patients with elevated HYS. The signature genes exhibited significant upregulation in GC tissues relative to para-carcinoma and normal gastric samples. Moreover, the hypoxia-related signature also correlated with distinct immune infiltration profiles and checkpoint expression, implying that immune suppression within the TME may contribute to the poor prognosis of high-risk HYS patients. In addition, our single-cell and pseudotime analyses indicated that hypoxia-related signature genes play important roles in the regulation of CAF development and differentiation in GC.

Collectively, these findings provide novel insights into the multifaceted role of hypoxia in GC progression. More importantly, our study provides additional evidence supporting the value of integrating bioinformatics analyses with immune landscape characterization, offering potential implications for prognostic stratification and the development of more effective immunotherapeutic strategies. Nevertheless, further validation using larger, independent cohorts and additional experimental studies is warranted to comprehensively elucidate the mechanisms linking hypoxia, immune regulation, and clinical outcomes in GC. Future work should focus on prospective validation in larger cohorts, systematic benchmarking against existing prognostic models, and mechanistic experiments to elucidate the roles of hypoxia-related genes in stromal–immune interactions and hypoxia-driven GC progression.

## Data Availability

The data used and/or analyzed in this study were obtained from the TCGA database and GEO database, which are available upon request and are publicly available databases.

## Supplementary Materials

The following supporting information can be downloaded at: https://www.mdpi.com/article/10.3390/biomedicines14020425/s1; Supplementary File S1. The significant markers in each cluster. Supplementary File S2. qRT-PCR primer sequences. Supplementary Figure S1. Independent validation of the hypoxia-related signature genes (*AXL*, *NRP1*, *SPARC*, and *VCAN*) using GC tissues from the TCGA cohort (n = 375) and normal gastric mucosa samples from the GTEx database (n = 359).

## Abbreviations

The following abbreviations are used in this manuscript:

GC	Gastric cancer
OS	Overall survival
HYS	Hypoxia-related gene signature
TME	Tumor microenvironment
ssGSEA	Single-sample gene set enrichment analysis
WGCNA	Weighted gene co-expression network analysis
GEO	Gene Expression Omnibus
TCGA	The Cancer Genome Atlas
scRNA-seq	Single-cell RNA sequencing
GO	Gene Ontology
KEGG	Kyoto Encyclopedia of Genes and Genomes
BP	Biological process
CC	Cellular component
MF	Molecular function
ROC	Receiver operating characteristic
AUC	Area under the curve
HR	Hazard ratio
CI	Confidence interval
K–M	Kaplan–Meier
LASSO	Least absolute shrinkage and selection operator
qRT-PCR	Quantitative real-time polymerase chain reaction
MM	Module membership
