# Supplementary material for: Integrated Weighted Gene Co-Expression Network and Single-Cell RNA Sequencing Analyses Reveal the Prognostic Significance of Hypoxia in Gastric Cancer
Source: Biomedicines. 2026 Feb 13;14(2):425. doi: 10.3390/biomedicines14020425 (PMC12937755; doi:10.3390/biomedicines14020425)
Supplement: Supplementary file 1 [file biomedicines-14-00425-s001.zip › Supplementary Figure S1.pdf]

Supplementary

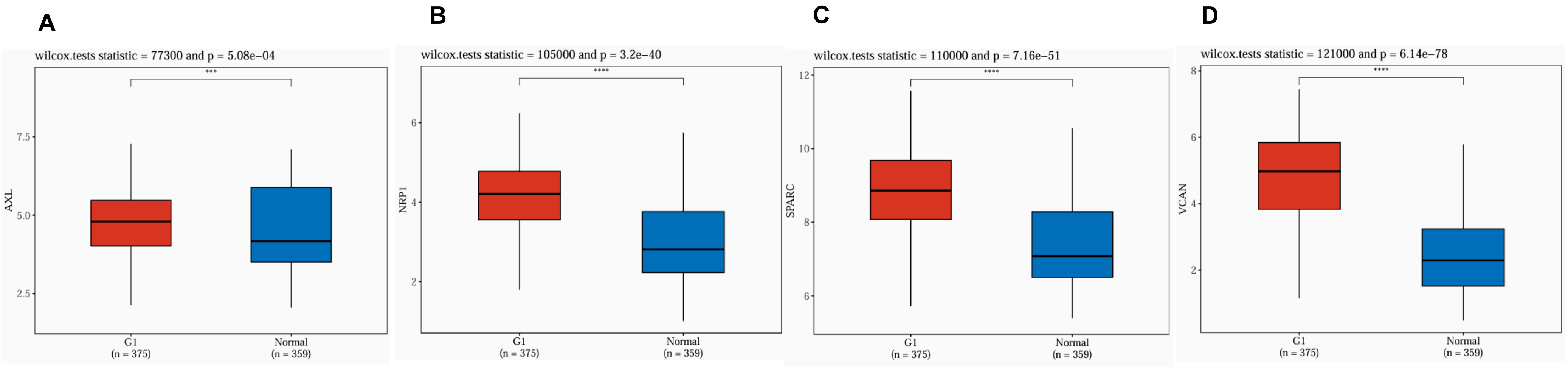

Figure S1. Independent validation of the hypoxia-related signature genes (*AXL*, *NRP1*, *SPARC*, and *VCAN*) using GC tissues from the TCGA cohort (n = 375) and normal gastric mucosa samples from the GTEx database (n = 359).
