# Supplementary material for: Integrated Weighted Gene Co-Expression Network and Single-Cell RNA Sequencing Analyses Reveal the Prognostic Significance of Hypoxia in Gastric Cancer
Source: Biomedicines. 2026 Feb 13;14(2):425. doi: 10.3390/biomedicines14020425 (PMC12937755; doi:10.3390/biomedicines14020425)
Supplement: Supplementary file 1 [file biomedicines-14-00425-s001.zip › Supplementary File S2.pdf]

Table S1. Primer sequences used for quantitative real-time PCR (RT-PCR)

Primer	Forward Sequences (5'-3')	Reverse Sequences (5'-3')
**SPARC**	TGGCGAGTTTGAGAAGGTGT	CAAGGCCCGATGTAGTCCAG
**AXL**	AGGTGGCTGTGAAGACGATG	CCCTGGAAACAGACACCGAT
**VCAN**	ATGGAGGAACGGCTTTGACC	TGCAGCGATCAGGTCGTTTA
**NPR1**	TGTGAAGTGGAAGCCCCTAC	CACCTGTGAGCTGGAAGTCA
